# Efficient preparation of 2-nitroimidazole nucleosides as precursors for hypoxia PET tracers

**DOI:** 10.1007/s00706-016-1874-8

**Published:** 2016-12-07

**Authors:** Petra Križková, Anna Wieczorek, Friedrich Hammerschmidt

**Affiliations:** Department of Organic Chemistry, University of Vienna, Vienna, Austria

**Keywords:** Hypoxia, 2-Deoxy-D-ribose nucleosides, 2-Nitroimidazole, Alkylation, Halogenides

## Abstract

**Abstract:**

2-Deoxy-D-ribose was converted to *α*/*β*-mixtures of methyl 3-*O*-acetyl- and methyl 3-*O*-benzoyl-2-deoxy-5-(*p*-toluenesulfonyl)-D-ribofuranosides. These were reacted with boron trichloride to generate ribofuranosyl chlorides, which afforded precursors for tracers to image tumor hypoxia on substitution with salts of 2-nitroimidazole. The anomeric ratio of the nucleosides was delicately influenced by the reaction conditions.

**Graphical abstract:**

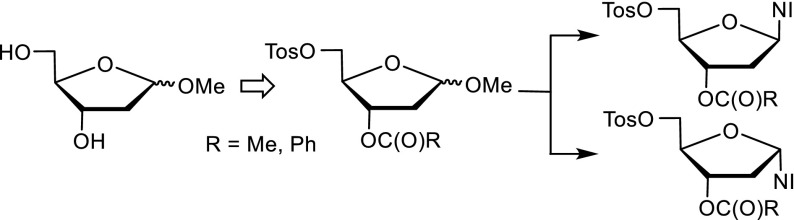

## Introduction

**Fig. 1:**
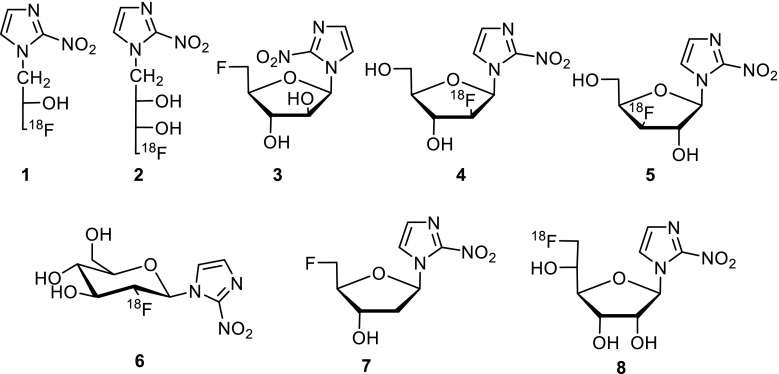
Known 2-nitroimidazole-based [^18^F]fluoro tracers

Tumor hypoxia has a negative prognosis predictive value for solid tumors, because it is associated with tumor aggressiveness, metastasis, and aberrant angiogenesis [1–3]. It reflects increased resistance to anticancer treatment by radio- and chemotherapy. Therefore, it is in the interest of cancer patients to identify and target hypoxic areas in solid tumors [4, 5]. Non-invasive in vivo quantification of hypoxic areas of solid tumors with radiolabeled tracers attracted much attention and was studied extensively in recent years [6, 7]. Fluorine-18 containing tracers derived from 2-nitroimidazole (azomycin) are the most important ones used for positron emission tomography (PET) to image hypoxia for diagnostic purposes. Under hypoxic conditions in cells, the 2-nitroimidazole moiety of the tracer is reduced stepwise by electron transfers via reactive intermediates [8, 9]. These attack low-molecular weight compounds, preferably glutathione, and to a lesser extent high molecular weight compounds, and the nitro group ends up as amino group. The modified compounds with the bound ^18^F, which is detected by PET, stay in the cells and are accumulated. Figure 1 is a compilation of those tracers, nucleosides derived from carbohydrates, such as various D-pentoses and D-hexoses, except compounds **1** and **2**. The first azomycin-based tracer and, at the same time, the gold standard up to now for imaging tumor hypoxia are [^18^F]fluoromisonidazole (FMISO, **1**) [7, 10]. A homologue thereof is [^18^F]fluoroerythronitroimidazole (**2**) [11]. From the [^18^F]fluoro nucleosides **3**-**8** derived from *α*-arabinose, tracer **3** [12, 13], from *β*-arabinose, tracer **4** [14], from *β*-xylose, tracer **5** [14], and from *β*-glucose, tracer **6** [15], only **3** gained prominence. Recently, we synthesized 2-nitroimadazole precursors derived from *α*- and *β*-2-deoxy-D-ribose and *α*- and *β*-D-allofuranose. The *β*-anomers were radiolabeled and deprotected to give tracers **7** [16] and **8** [17] so far and evaluated for imaging tumor hypoxia.

## Results and discussion

The synthesis of the precursors for tracers *α*- and *β*-**8** is given in Scheme 1 [16]. In brief, it started from 2-deoxy-D-ribose, which was converted via methyl glycosides **10** to fully protected methyl glycosides **11**. Their mixture was treated with 8 M HCl/Et_2_O/CH_2_Cl_2_ at 0 °C to form a mixture of glycosyl chlorides which was reacted with the tetrabutylammonium salt of 2-nitroimidazole. The two nucleosides, *α*- and *β*-**12**, were separated by flash column chromatography and individually desilylated and finally tosylated to give the two desired precursors *α*- and *β*-**14**. This sequence was selected, because we thought that introduction of the tosyl group right from the beginning would not be tolerated by 8 M HCl in Et_2_O/CH_2_Cl_2_. However, if that worked, the synthesis of both precursors could be shortened. Furthermore, we wanted to replace the tedious preparation of 8 M HCl in Et_2_O by a commercially available and more reactive reagent, such as BCl_3_, for the conversion of the methyl glycosides into the glycosyl chlorides.

**Figure:**
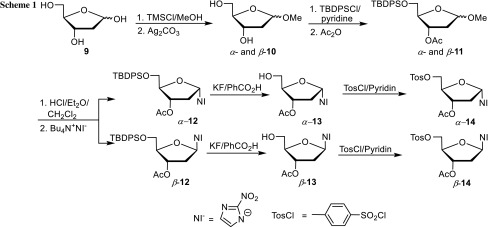

The improved synthesis is given in Scheme 2. Although the mixture of methyl glycosides *α*- and *β*-**10** [18] was tosylated [19] at −25 °C for 3 days in 59% yield (*α*/*β* = 1.2/1), some ditosylate **16** was formed as well (11%, *α*/*β* = 1.4/1). Analytical samples of the anomers for characterization could not be obtained by column flash chromatography. However, they could be obtained in homogeneous form by deacetylation of (+)- and (−)-**17** and allowed to assign the anomeric configuration as will be shown later. Acetylation of the mixture of tosylates *α*- and *β*-**15** with Ac_2_O in dry pyridine delivered a mixture of acetates *α*- and *β*-**17** in 92% (*α*/*β* = 1.2/1) yield. This mixture could be separated by flash column chromatography and Zemplen saponification of acetates (+)- and (−)-**17** delivered homogenous samples of *α*- and *β*-**15**, respectively. The latter one is a literature known compound whose *β*-configuration has been determined by 2D NMR methods [20]. It allowed to assign *α*-configuration to (+)-**15** and *α* and *β* to (+)- and (−)-**17**, respectively. As glycosides *α*- and *β*-**17** were less reactive with HCl/Et_2_O in CH_2_Cl_2_, BCl_3_ in CH_2_Cl_2_ (1 M) was found to be an alternative to generate the glycosyl chlorides at 0 °C (general procedure A). Rapid aqueous work up at 0 °C allowed to isolate the labile chlorides, which were immediately reacted in two ways with 2-nitroimidazole. In the first case (general procedure B), the tetrabutylammonium salt of 2-nitroimidazole [21] was mixed with a solution of the 2-deoxy-D-ribofuranosyl chloride at −30 °C in CH_2_Cl_2_. The reaction mixture was allowed to warm slowly to 0 °C within 2 h and was then extractively worked up. Flash chromatography furnished known anomers *α*- and *β*-**14** over two steps in 41 and 11% yield, respectively. When the reaction was started at −50 °C, the *α*/*β*-**14** ratio was 5/1 (by NMR) and only the *α*-anomer was isolated in 53% yield. In the second case (general procedure C), the mixture of glycosyl chlorides was added to a mixture of 2-nitroimidazole/K_2_CO_3_/excess tris[2-(2-methoxyethoxy)ethyl]amine (TDA-1) as phase transfer catalyst [22] in CH_3_CN at 0 °C. Work up after 2 h and purification delivered 12% of nucleoside *α*-**14** and 36% of *β*-**14** starting from methyl glycosides. Satisfyingly, the two complementary procedures gave either preferably *α*- or *β*-anomer **14** [16].

**Figure:**
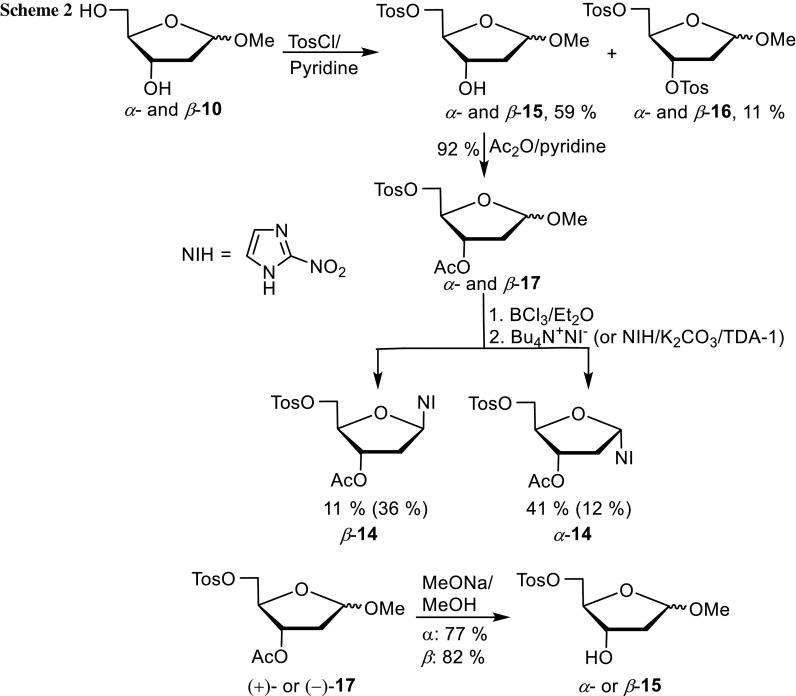

We aimed to increase the yields of the nucleosides by replacing the acetyl protecting group by the more stable benzoyl group (Scheme 3). Therefore, the mixture of tosylates *α*- and *β*-**15** was benzoylated and gave again a mixture of globally protected 2-deoxy-D-riboses *α*- and *β*-**18**, which could not be separated by flash column chromatography to obtain homogeneous analytical samples. Benzoylation of alcohols *α*- and *β*-**15** with benzoyl chloride/pyridine affords the individual anomers of **18** for analytical purposes, although the mixture was used for the next step. It was converted to chlorides as before with BCl_3_ in CH_2_Cl_2_ according to general procedure A. Their isolation without purification was immediately followed by reaction with the tetrabutylammonium salt of 2-nitroimidazole, starting the reaction at −50 °C and allowing it to warm to ambient temperature. The mixture of the nucleosides *α*- and *β*-**19** was isolated in 81% yield (*α*/*β* = 2/1) by flash chromatography. The individual anomers were obtained by a second flash chromatography. When general procedure C was used for the preparation of the nucleosides from the chlorides, the yield of *α*-**19** was 14% and that of *β*-**19** was 69%. As envisioned, the yields with the benzoyl protecting group were higher than with the acetyl version. The anomeric configuration of *α*- and *β*-**19** (^1^H NMR; *α*-1-H′: d, *J* = 6.6 Hz; *β*-1-H′: dd, *J* = 7.5 and 5.6 Hz) was assigned in analogy to nucleosides *α*- and *β*-**14** (^1^H NMR; *α*-1-H′: dd, *J* = 6.3, 0.8 Hz; *β*-1-H′: dd, *J* = 6.6, 6.1 Hz) and the literature known analogue [19] of *β*-**14** with two 4-toluoyl protecting groups (^1^H NMR; *β*-1-H′: t, *J* = 6.5 Hz) instead of the acetyl and benzoyl group. The 1-H′ hydrogen atoms of the *α*-anomers resonate as doublets or as doublets of doublets with one coupling constant being very small. However, the 1-H′ hydrogen atoms of the *β*-anomers resonate as doublets of doublets or as triplet with two similar coupling constants.

**Figure:**
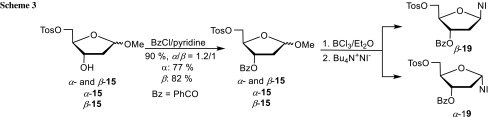

## Conclusions

The synthesis of known 2-nitroimidazole nucleosides derived from 2-deoxy-D-ribose used as precursors for tracers was shortened if tosylation is performed at the beginning instead of at the end of the reaction sequence. The yield was further improved using BCl_3_ for generation of 2-deoxy-D-ribofuranosyl chlorides and benzoyl instead of acetyl group as protecting group for OH at C-3.

## Experimental

^1^H, ^13^C (*J*-modulated; not *J*-modulated spectra were recorded of 2-nitroimidazole derivatives) NMR spectra were recorded in CDCl_3_ on a Bruker AV III 400 (^1^H: 400.27 MHz, ^13^C: 100.65 MHz), AV 400 (^1^H: 400.13 MHz, ^13^C: 100.61 MHz), and AV III 600 (^1^H: 600.13 MHz, ^13^C: 150.90 MHz) spectrometer at 25 °C, respectively. Chemical shifts *δ* (ppm) were referenced to residual CHCl_3_ (*δ*_H_ = 7.24 ppm) and CDCl_3_ (*δ*_C_ = 77.00 ppm). IR spectra were recorded on a Bruker VERTEX 70 IR spectrometer as ATR spectra or of films on a silicon disc [23] on a Perkin Elmer 1600 FT-IR spectrometer. Optical rotations were measured at 20 °C on a Perkin Elmer 351 polarimeter in a 10 cm cell. Melting points were determined on a Reichert Thermovar instrument. Elemental analyses (C, H, N, S) were conducted using the Euro EA 3000 Elemental Analyser (for oxygen in combination with a high temperature pyrolysis furnace (1480 °C) and reduction with carbon) from Eurovector. Their results were found to be in good agreement (±0.3%) with the calculated values.

Flash (column) chromatography was performed with Merck silica gel 60 (230–400 mesh). TLC was carried out on 0.25 mm-thick Merck plates, silica gel 60 F_254_. Spots were visualized by UV and/or dipping the plate into a solution of 23.0 g (NH_4_)_6_Mo_7_O_24_·4H_2_O and 1.0 g Ce(SO_4_)_2_·4H_2_O in 500 cm^3^ 10% aqueous H_2_SO_4_, followed by heating with a heat gun. Pyridine was dried by refluxing over powdered CaH_2_, then distilled and stored over molecular sieves (4 Å). Dichloromethane was dried by storage over molecular sieves (3 Å). All other chemicals and solvents were of the highest purity available and used as received.

### *Mixture of methyl 2*-*deoxy*-*5*-*O*-*(p*-*toluenesulfonyl)*-*α*- *and methyl 2*-*deoxy*-*5*-*O*-*(p*-*toluenesulfonyl)*-*β*-*D*-*ribofuranoside* (*α*- and *β*-**15**, C_13_H_18_O_6_S) *and mixture of methyl 3,5*-*bis(p*-*toluenesulfonyl)*-*α*- *and methyl 3,5*-*bis(p*-*toluenesulfonyl)*-*β*-*D*-*ribofuranoside* (*α*- and *β*-**16**, C_20_H_24_O_8_S_2_)

Dry pyridine (2.20 cm^3^, 27.24 mmol) was added to a mixture of 1.345 g methyl glycosides *α*- and *β*-**10** (9.08 mmol) [18] in 17 cm^3^ dry CH_2_Cl_2_ under Ar. The stirred reaction mixture was cooled to 0 °C and 1.868 g *p*-toluenesulfonyl chloride (9.08 mmol) was added. The flask was stored at −25 °C for 3 days and afterwards 1 cm^3^ water was added. After stirring for 15 min, the reaction mixture was concentrated under reduced pressure and 10 cm^3^ EtOAc was added. The organic phase was washed with 10 cm^3^ 2 M HCl, 10 cm^3^ water, and 10 cm^3^ NaHCO_3_, then dried (Na_2_SO_4_) and concentrated under reduced pressure. The residue was purified by flash chromatography (hexanes/EtOAc = 1/1; *R*_*f*_ = 0.34 for monotosylates, *R*_*f*_ = 0.75 for ditosylates) giving 1.631 g mixture of monotosylates *α*- and *β*-**15** (59%; *α*/*β* = 1.22/1) and 0.458 g mixture of ditosylates *α*- and *β*-**16** (11%), both as colorless oils. The data of the individual anomers are given later. Mixture of ditosylates *α*- and *β*-**16**: [*α*]_D_^20^ = + 24.3 cm^2^ g^−1^ (*c* = 1.55, acetone); IR (ATR, NMR sample in CDCl_3_): $\bar{\nu }$ = 1359, 1190, 1174, 1096, 976 cm^−1^.

^1^H NMR (400.27 MHz, CDCl_3_): *α*/*β* = 1.4/1.0; contained 5% by weight of toluene; *α*-**16**: *δ* = 7.76–7.71 (m, 4H, H^Ar^), 7.36–7.30 (m, 4H, H^Ar^), 4.92 (bd, *J* = 5.2 Hz, 1H, 1-H), 4.77 (ddd, *J* = 8.3, 3.4, 2.0 Hz, 1H, 3-H), 4.29 (q, *J* = 3.4 Hz, 1H, 4-H), 4.09 (d, *J* = 3.4 Hz, 2H, 5-H), 3.27 (s, 3H, OCH_3_), 2.43 (s, 6H, CH_3_^tol^), 2.13 (ddd, *J* = 14.8, 8.3, 5.2 Hz, 1H, 2-H), 1.96 (ddd, *J* = 14.8, 2.0. 0.8 Hz, 1H, 2-H) ppm; *β*-**16**: *δ* = 7.76–7.71 (m, 4H, H^Ar^), 7.36–7.30 (m, 4H, H^Ar^), 5.00 (dd, *J* = 4.9, 2.6 Hz, 1H, 1-H), 4.88 (ddd, *J* = 9.0, 5.9 Hz, 1H, 3-H), 4.18 (td, *J* = 5.9, 3.5 Hz, 1H, 4-H), 3.92 (AB part of ABX system, *J*_AB_ = 10.4 Hz, *J*_AX_ = *J*_BX_ = 5.9 Hz, 2H, 5-H), 3.18 (s, 3H, OCH_3_), 2.43 (s, 6H, CH_3_^tol^), 2.20–2.15 (m, 2H, 2-H) ppm; ^13^C NMR (100.65 MHz, CDCl_3_) of mixture: *δ* = 145.4 (Cq, *β*), 145.2(Cq, *α*), 145.0 (Cq, *α*), 145.0 (Cq, *β*), 133.0 (Cq, *α*), 132.8 (Cq, *β*), 132.5 (Cq, *β*), 132.5 (Cq, *α*), 130.1 (2 CH, *β*), 130.0 (2 CH, *α*), 129.8 (4 CH, *α* and *β*), 127.9 (4 CH), 127.8 (2 CH), 127.8 (2 CH), 105.1 (C-1, *β*), 104.7 (C-1, *α*), 80.5 (C-4, β), 80.4 (C-4, *α*), 80.1 (C-3, *β*), 79.1 (C-3, *α*), 68.8 (C-5, *β*), 68.4 (C-5, *α*), 55.1 (CH_3_O, *β*), 64.99 (OCH_3_, α), 39.0 (C-2, *β*), 38.9 (C-2, *α*), 21.6 (2 CH_3_), 21.55 (2 CH_3_) ppm.

### *Mixture of (*+*)*- *and (*−*)*-*methyl 3*-*O*-*acetyl*-*2*-*deoxy*-*5*-*O*-*(p*-*toluenesulfonyl)*-*D*-*ribofuranoside* ((+)- and (−)-**17**, C_15_H_20_O_7_S)

To 1.631 g mixture of monotosylates *α*- and *β*-**5** (5.39 mmol), 1.02 cm^3^ Ac_2_O (10.78 mmol) and 1.67 cm^3^ dry pyridine (21.56 mmol) in 13 cm^3^ dry CH_2_Cl_2_ were added under Ar. The reaction mixture was heated at 40 °C until the starting material was consumed (about 4 h). After addition of 4 cm^3^ water, stirring was continued for 15 min. The organic phase was separated and washed with 15 cm^3^ 2 M HCl and 15 cm^3^ saturated aqueous solution of NaHCO_3_, dried (MgSO_4_), and concentrated under reduced pressure. The residue was purified by flash chromatography (hexanes/EtOAc = 1/1, *R*_*f*_ = 0.82, 0.75 for anomers) to yield 1.704 g mixture of anomers (92%, *α*/*β* = 1.2/1.0) as colorless oil. Part of the mixture was flash chromatographed to get analytical samples of anomers (+)- and (−)-**17** (less polar isomer: *R*_*f*_ = 0.20, more polar isomer: *R*_*f*_ = 0.11 for hexanes/EtOAc = 3/1) as colorless oils.

(−)-**17**: *R*_*f*_ = 0.20 (hexanes/EtOAc = 3/1); colorless crystals, m.p.: 50–51 °C (*i*-Pr_2_O/hexanes); [*α*]_D_^20^ = −40.7 cm^2^ g^−1^ (*c* = 1.01, acetone); ^1^H NMR (600.13 MHz, CDCl_3_): *δ* = 7.81–7.78 (m, 2H, H^tos^), 7.34–7.30 (m, 2H, H^tos^), 5.08 (ddd, *J* = 7.3, 5.3, 2.3 Hz, 1H, 3-H), 5.04 (dd, *J* = 5.4, 2.0 Hz, 1H, 1-H), 4.19–2.13 (m, 2H, 4-H and 5-H), 4.04 (dd, 9.7, 6.6 Hz, 1H, 5-H), 3.21 (s, 3H, OCH_3_), 2.42 (s, 3H, CH_3_^tos^), 2.31 (ddd, *J* = 14.0, 7.3, 2.0 Hz, 1H, 2-H), 2.09 (d, *J* = 14.0, 5.3 Hz, 1H, 2-H), 2.01 (s, 3H, CH_3_CO) ppm; ^13^C NMR (150.90 MHz, CDCl_3_): *δ* = 171.0 (C=O), 144.9 (Cq^tos^), 132.8 (Cq^tos^), 129.8 (2 CH), 127.9 (2 CH), 105.2 (C-1), 80.9 (C-4), 74.0 (C-3), 69.5 (C-5), 55.2 (OCH_3_), 38.8 (C-2), 21.6 (CH_3_^tos^), 21.0 (CH_3_) ppm; and IR (ATR): $\bar{\nu }$ = 2925, 1737, 1360, 1235, 1175, 1047, 973, 955 cm^−1^.

(+)-**17**: *R*_*f*_ = 0.11 (hexanes/EtOAc = 3/1), oil; [*α*]_D_^20^ = +93.6 (*c* = 1.05, acetone); ^1^H NMR (600.13 MHz, CDCl_3_): *δ* = 7.79–7.75 (m, 2H, H^tos^), 7.35–7.30 (m, 2H, H^tos^), 4.98 (dd, *J* = 5.3, 0.7 Hz, 1H, 1-H), 4.94 (ddd, *J* = 8.3, 3.5, 1.9 Hz, 1H, 3-H), 4.21 (AB part of ABX system, *J*_AB_ = 10.6 Hz, *J*_4,5_ = 3.5 and 3.2 Hz, 2H, 5-H), 4.15 (~q, *J* = ~3.5 Hz, 1H, 4-H), 3.31 (s, 3H, OCH_3_), 2.42 (s, 3H, CH_3_ tol), 2.28 (ddd, *J* = 14.5, 8.3, 5.3 Hz, 1H, 2-H), 2.02 (s, 3H, CH_3_CO), 1.95 (ddd, *J* = 14.5, 1.9, 0.7 Hz, 1H, 2-H) ppm; ^13^C NMR (150.90 MHz, CDCl_3_): *δ* = 171.0 (C=O), 144.9 (Cq^tos^), 132.8 (Cq^tos^), 129.8 (2 CH), 127.9 (2 CH), 105.2 (C-1), 80.9 (C-4), 74.0 (C-3), 69.5 (C-5), 55.2 (OCH_3_), 38.8 (C-2), 21.6 (CH_3_^tos^), 21.0 (CH_3_) ppm; and IR (ATR): $\bar{\nu }$ = 2836, 1736, 1364, 1240, 1177, 1070, 1020, 978 cm^−1^.

### *Methyl 2*-*deoxy*-*5*-*O*-*(p*-*toluenesulfonyl)*-*α*-*D*-*ribofuranoside* (*α*-**15**, C_13_H_18_O_6_S)

A solution of 0.291 g acetate (+)-**17** (0.84 mmol, [*α*]_D_^20^ = +93.6 (*c* = 1.05, acetone)), 4.25 cm^3^ dry MeOH, and 0.43 cm^3^ NaOMe/MeOH (0.425 mmol, 1 M) was stirred for 30 min at 0 °C (TLC). Dry ice was added to neutralize base. The reaction mixture was concentrated under reduced pressure. Water (10 cm^3^) and 5 cm^3^ CH_2_Cl_2_ were added. The organic phase was separated and the aqueous one extracted with CH_2_Cl_2_ (2 × 5 cm^3^). The combined organic layers were dried (Na_2_SO_4_) and concentrated under reduced pressure. The residue was purified by flash chromatography (hexanes/EtOAc = 1/1, *R*_*f*_ = 0.47) to yield 0.199 g alcohol *α*-**15** (77%) as colorless oil. [*α*]_D_^20^ = + 95.09° g cm^2^ (*c* = 1.12, acetone); ^1^H NMR (400.13 MHz, CDCl_3_): *δ* = 7.78–7.73 (m, 2H, H^tos^), 7.35–7.30 (m, 2H, H^tos^), 5.01 (d, *J* = 4.4 Hz, 1H, 1-H), 4.18 (≈td, *J* = 4.1, 1.8 Hz, 1H, 4-H), 4.10 (bd, *J* = 5.9 Hz, 1H, 3H), 4.04 (AB part of ABX system, *J*_AB_ = 10.7 Hz, *J* = 4.3, 3.8 Hz, 2H, 5-H), 3.32 (s, 3H, CH_3_O), 2.75 (bs, 1H, OH), 2.43 (s, 3H, CH_3_), 2.05 (ddd, *J* = 13.9, 5.9, 4.4 Hz, 1H, 2-H), 1.96 (dd, *J* = 13.9, 0.8 Hz, 1H, 2-H) ppm; ^13^C NMR (100.61 MHz, CDCl_3_): *δ* = 145.0 (Cq, CSO_3_), 132.7 (Cq^tos^), 129.90 (2 HC^tos^), 127.9 (2 HC^tos^), 105.7 (C-1), 84.6 (C-4), 72.8 (C-3), 69.4 (C-5), 55.0 (OCH_3_), 41.0 (C-2), 21.6 (CH_3_^tos^) ppm; and IR (Si): $\bar{\nu }$ = 3445, 2923, 1354, 1173, 1081, 961, 908 cm^−1^.

### *Methyl 2*-*deoxy*-*5*-*O*-*(p*-*toluenesulfonyl)*-*β*-*D*-*ribofuranoside* (*β*-**15**, C_13_H_18_O_6_S)

A mixture of 0.066 g acetate (−)-**17** (0.19 mmol, less polar acetate, [*α*]_D_^20^ = −40.7 cm^2^ g^−1^ (*c* = 1.01, acetone)), 2 cm^3^ dry MeOH, and 0.064 cm^3^ MeONa/MeOH (0.064 mmol, 0.33 equiv, 1 M) was stirred at -30 °C. The ester was consumed after 5 h (TLC). Work up as for *α*-**15** yielded 0.047 g alcohol *β*-**15** (82%) as colorless oil. [*α*]_D_^20^ = −40.4 g cm^2^ (*c* = 1.08, acetone); ^1^H NMR (400.13 MHz, CDCl_3_): *δ* = 7.81–7.74 (m, 2H, H^tos^), 7.36–7.31 (m, 2H, H^tos^), 5.01 (dd, *J* = 5.2, 1.8 Hz, 1H, 1-H), 4.41 (bs, 1H, 3-H), 4.07–3.99 (m, 3H, 5-H, 4-H), 3.20 (s, 3H, OCH_3_), 2.43 (s, 3H, CH_3_^tos^), 2.19 (ddd, *J* = 13.4, 6.9, 1.8 Hz, 1H, 2-H), 2.10 (bs, 1H, OH), 2.03 (ddd, *J* = 13.4, 6.2, 5.2 Hz, 1H, 2-H) ppm; ^13^C NMR (100.61 MHz, CDCl_3_): *δ* = 145.1 (Cq, CSO_3_), 132.7 (Cq, C^tos^), 129.9 (2C, HC^tos^), 128.0 (2C, HC^tos^), 105.3 (C-1), 82.9 (C-4), 72.65 (C-3), 70.14 (C-5), 50.02 (CH_3_O), 41.32 (C-2), 21.64 (CH_3_ tol) ppm.

### *Preparation of anomeric 3*-*O*-*acetyl*-*2*-*deoxy*-*5*-*O*-*(p*-*toluenesulfonyl)*-*D*-*ribofuranosyl chlorides (general procedure A) and their conversion to 1*-*(3′*-*O*-*acetyl*-*2′*-*deoxy*-*5′*-*O*-*(p*-*toluenesulfonyl)*-*α*- *and 1*-*(3′*-*O*-*acetyl*-*2′*-*deoxy*-*5′*-*O*-*(p*-*toluenesulfonyl)*-*β*-*D*-*ribofuranosyl)*-*2*-*nitroimidazole (α*- *and β*-***14****)*

General procedure A: To a solution of 0.507 g methyl glycosides, *α*- and *β*-**17** (1.47 mmol) in 4.5 cm^3^ dry Et_2_O at 0 °C under Ar 3.68 cm^3^ BCl_3_ (3.68 mmol, 2.5 equiv, 1 M in CH_2_Cl_2_) was added. The reaction mixture was stirred for 2 h (TLC: hexanes/EtOAc = 1/1; virtually no starting material was present; new strong spot with *R*_*f*_ = 0.34) at 0 °C. CH_2_Cl_2_ (12 cm^3^, 0 °C) was added and the mixture was washed with 4 cm^3^ cold brine (−18 °C), which was then extracted with 5 cm^3^ cold CH_2_Cl_2_ (0 °C). The combined organic phases were washed with 5 cm^3^ cold aqueous solution of NaHCO_3_ (0 °C), dried (Na_2_SO_4_) at 0 °C, and concentrated first to 5–10 cm^3^ on a rotavapor without warming with the water bath and then the remaining solvent was removed on the vacuum pump (1 mbar) within a few min without warming. The clear somewhat coloured solution was used immediately for the next step after withdrawing a sample for ^1^H NMR spectroscopy; ratio of chlorides: *α*/*β* = 3.6/1.0.

^1^H NMR of anomeric 2-deoxy-D-ribofuranosyl chlorides (400.27 MHz, CDCl_3_): *δ* = 6.21 ppm (d, *J* = 5.3 Hz, 1-H of *α*-chloride), 1-H of *β*-chloride overlapping with 1-H of *α*-chloride, integration was referenced to resonance at 4.46 ppm (q, *J* = 2.9 Hz, 4-H).

### *Reaction of anomeric 2*-*deoxy*-*D*-*ribofuranosyl chlorides with tetrabutylammonium salt of 2*-*nitroimidazole (general procedure B)*

A solution of the above 2-deoxy-D-ribofuranosyl chlorides derived from *α*- and *β*-**17** in 3.5 cm^3^ dry CH_2_Cl_2_ (0 °C) was added to a solution of the 0.450 g tetrabutylammonium salt of 2-nitroimidazole (1.32 mmol, 0.9 equiv. relative to methyl glycosides) [21] in dry 4 cm^3^ CH_2_Cl_2_ at −30 °C under Ar. Stirring was continued for 2 h, while the cooling bath was allowed to reach 0 °C. The reaction mixture was concentrated under reduced pressure. The residue was dissolved in 15 cm^3^ EtOAc and washed with water (2 × 5 cm^3^). The organic phase was dried (MgSO_4_) and concentrated under reduced pressure. The residue (*α*/*β* = 2/1 by ^1^H NMR) was flash chromatographed (hexanes/EtOAc = 1/1, *α*: *R*_*f*_ = 0.29; *β*: *R*_*f*_ = 0.49) to yield 0.060 g *β*-**14** (11%) and 0.231 g *α*-**14** (41%), both spectroscopically (^1^H, ^13^C NMR) identical to the ones described in Ref. [14].

Similarly, 0.536 g mixture of anomeric methyl glycosides (1.56 mmol) were converted via chlorides to nucleosides (reaction was started at −50 °C); ratio of *α*/*β* = 5/1 by ^1^H NMR in crude product. Flash chromatography furnished 0.318 g *α*-**14** (53%).

### *Reaction of 3*-*O*-*acetyl*-*glycosyl chlorides with 2*-*nitroimidazole/K*_*2*_*CO*_*3*_*/tris[2*-*(2*-*methoxyethoxy)ethyl]amine (TDA*-*1) (general procedure C)*

A mixture of 0.118 g 2-nitroimidazole (1.04 mmol, 0.8 equiv.), 0.225 g K_2_CO_3_ (1.63 mmol), 10 mm^3^ TDA-1 [23], and 20 cm^3^ dry CH_3_CN was stirred for 10 min at RT under Ar and then cooled to 0 °C. The chlorides prepared from 0.449 g methyl glycosides *α*- and *β*-**17** (1.30 mmol) by the above given general procedure A were dissolved in dry CH_3_CN at 0 °C and added. Stirring was continued for 2 h at 0 °C and then the reaction mixture was filtered through Celite (washing with CH_2_Cl_2_). The filtrate was concentrated under reduced pressure and 20 cm^3^ EtOAc was added to the residue. The mixture was washed with water (2 × 10 cm^3^), dried (MgSO_4_), and concentrated under reduced pressure. The residue (*α*/*β* = 1/3, by ^1^H NMR) was purified by flash chromatography (hexanes/EtOAc = 1/1) to yield 54 mg *α*-**14** (12%) and 160 mg *β*-**14** (36%).

### *Mixture of methyl 3*-*O*-*benzoyl*-*2*-*deoxy*-*5*-*O*-*(p*-*toluenesulfonyl)*-*α*- *and methyl 3*-*O*-*benzoyl*-*2*-*deoxy*-*5*-*O*-*(p*-*toluenesulfonyl)*-*β*-*D*-*ribofuranoside* (*α*- and *β*-**18**, C_20_H_22_O_7_S)

To 0.800 g, mixture of anomeric monotosylates **15** (2.65 mmol) and 0.64 cm^3^ dry pyridine (7.95 mmol) in dry CH_2_Cl_2_ (6.3 cm^3^) under Ar was added 0.64 cm^3^ benzoyl chloride. The reaction mixture was stirred at RT for 18 h. After addition of 0.5 cm^3^ water, stirring was continued for 15 min. The mixture was concentrated under reduced pressure and 15 cm^3^ EtOAc and 5 cm^3^ water were added. The organic phase was separated and washed with 5 cm^3^ 2 M HCl, 5 cm^3^ water, and 5 cm^3^ saturated aqueous solution of NaHCO_3_, dried (MgSO_4_), and concentrated under reduced pressure. The residue was purified by flash chromatography (hexanes/EtOAc = 2/1, *R*_*f*_ = 0.76) to yield 0.972 g mixture of anomeric benzoates **18** (90%; *α*/*β* = 1.2/1.0 by ^1^H NMR) as a colorless oil possibly containing some benzoic acid; [*α*]_D_^20^ = + 41.0 g cm^2^ (*c* = 1.35, acetone). The individual anomers of **18** for characterization were prepared by esterification of homogeneous anomers *α*- and *β*-**15** with PhC(O)Cl/pyridine.

### *Methyl 3*-*O*-*benzoyl*-*2*-*deoxy*-*5*-*O*-*(p*-*toluenesulfonyl)*-*α*- *and methyl 3*-*O*-*benzoyl*-*2*-*deoxy*-*5*-*O*-*(p*-*toluenesulfonyl)*-*β*-*D*-*ribofuranoside* (*α*- and *β*-**18**, C_20_H_22_O_7_S)

Benzoyl chloride (0.143 g, 1.02 mmol, 0.118 cm^3^) and 0.120 g dry pyridine (1.52 mmol, 0.122 cm^3^) were added to 0.153 g alcohol *α*-**15** (0.51 mmol) dissolved in 1.5 cm^3^ dry CH_2_Cl_2_ and the solution was stirred for 20 h at RT. Water (0.5 cm^3^) was added and the reaction mixture was stirred for 15 min. The mixture was concentrated under reduced pressure, 10 cm^3^ water was added, and it was extracted with ethyl acetate (3 × 5 cm^3^), dried with Na_2_SO_4_, and concentrated under reduced pressure. The crude product was purified by flash chromatography (hexanes/ethyl acetate = 1/1, *R*_*f*_ = 0.55) to yield 0.183 g benzoate *α*-**18** (88%) as colorless oil. Similarly, 0.096 g alcohol *β*-**15** (0.32 mmol) was converted to 0.104 g benzoate *β*-**18** (81%).

*α*-**18**: [*α*]_D_^20^ = + 98.23 (*c* = 1.015, acetone); ^1^H NMR (400.13 MHz, CDCl_3_): *δ* = 8.01–7.96 (m, 2H, H^Ph^), 7.81–7.76 (m, 2H, H^tos^), 7.58–7.52 (m, 1H, H^Ph^), 7.45–7.38 (m, 2H, H^Ph^), 7.34–7.28 (m, 2H, H^tos^), 5.22–5.16 (m, 1H, 3-H), 5.06 (d, *J* = 5.2 Hz, 1H, 1-H), 4.35–4.27 (m, 3H, 4-H, 5-H), 3.34 (s, 3H, CH_3_O), 2.41 (s, 3H, CH_3_^tos^), 2.40 (ddd, *J* = 14.5, 8.1, 5.2 Hz, 1H, 2-H), 2.11 (dd, *J* = 14.5, 1.5 Hz, 1H, 2-H) ppm; ^13^C NMR (100.61 MHz, CDCl_3_): *δ* = 166.41 (CO), 144.92 (CSO_3_), 133.27 (HC^Ph^), 132.86 (CH_3_ *C* ^tos^), 129.86 (2C, HC^tos^), 129.73 (2C, HC^Ph^), 129.58 (*C*CO), 128.39 (2C, HC^ar^), 127.99 (2C, HC^ar^), 105.26 (C-1), 80.945 (C-4), 74.49 (C-3), 69.56 (C-5), 55.14 (CH_3_O), 38.90 (C-2), 21.62 (CH_3_^tos^) ppm; and IR (Si): $\bar{\nu }$ = 3016, 2970, 2946, 1738, 1725, 1365, 1229, 1217 cm^−1^.

*β*-**18**: [*α*]_D_^20^ = −16.75 (*c* = 0.83, acetone); ^1^H NMR (400.13 MHz, CDCl_3_): *δ* = 7.98–7.93 (m, 2H, H^Ph^), 7.83–7.77 (m, 2H, H^tos^), 7.59–7.53 (m, 1H, H^Ph^), 7.46–7.38 (m, 2H, H^Ph^), 7.33–7.27 (m, 2H, H^tos^), 5.32 (ddd, *J* = 7.5, 5.4, 3.3 Hz, 1H, 3-H), 5.13 (dd, *J* = 5.4, 2.0, Hz, 1H, 1-H), 4.32 (ddd, *J* = 7.1, 5.1, 3.3 Hz, 1H, 4-H), 4.26 (dd, *J* = 10.1, 5.1 Hz, 1H, 5-H), 4.14 (dd, *J* = 10.1, 7.1 Hz, 1H, 5-H), 3.26 (s, 3H, OCH_3_), 2.43 (ddd, *J* = 14.2, 7.3, 2.0 Hz, 1H, 2-H), 2.39 (s, 3H, CH_3_^tos^), 2.25 (td, *J* = 14.2, 5.4 Hz, 1H, 2-H) ppm; ^13^C NMR (100.61 MHz, CDCl_3_): *δ* = 165.95 (CO), 144.90 (CSO_3_), 133.38 (HC^Ph^), 132.84 (CH_3_ *C* ^tos^), 129.86 (2C, HC^tos^), 129.64 (2C, HC^Ph^), 129.35 (*C*CO), 128.44 (2C, HC^Ph^), 128.02 (2C, HC^tos^), 105.70 (C-1), 81.39 (C-4), 75.10 (C-3), 70.35 (C-5), 55.19 (CH_3_O), 39.07 (C-2), 21.60 (CH_3_^tos^) ppm; and IR (Si): $\bar{\nu }$ = 2924, 1721, 1365, 1274, 1178, 1110 cm^−1^.

### *Preparation of mixture of anomeric 3*-*O*-*benzoyl*-*2*-*deoxy*-*5*-*O*-*(p*-*toluenesulfonyl)*-*D*-*ribofuranosyl chlorides and their conversion to 1*-*(3′*-*O*-*benzoyl*-*2′*-*deoxy*-*5′*-*O*-*(p*-*toluenesulfonyl)*-*α*- *and 3′*-*O*-*benzoyl*-*2′*-*deoxy*-*5′*-*O*-*(p*-*toluenesulfonyl)*-*β*-*D*-*ribofuranosyl)*-*2*-*nitroimidazole* (*α*- and *β*-**19**, C_22_H_21_N_3_O_8_S)

A mixture of 0.609 g methyl glycosides *α*- and *β*-**19** (1.50 mmol) was converted to 3-*O*-benzoyl-glycosyl chlorides (TLC: hexanes/EtOAc = 1/1, *R*_*f*_ = 0.58) by the procedure used for methyl glycosides α- and *β*-**17** (general procedure A). The crude product was used immediately for the next step. ^1^H NMR spectrum of crude 3-benzoyl 2-deoxy-D-ribofuranosyl chlorides (400.27 MHz, CDCl_3_): *δ* = 6.31 (d, *J* = 5.0 Hz, 1-H of *α*-chloride), 6.28 (dd, *J* = 5.5, 1.6 Hz, 1-H of *β*-chloride), integration referenced to resonance at 4.60 ppm (q, *J* = 2.6 Hz, 4-H); *α*/*β* = 1/0.13; fairly pure.

The above mixture of chlorides was converted to a mixture of *α*- and *β*-**19** using the procedure (general procedure B) given for the corresponding 3-*O*-acetyl-glycosyl chlorides. Tetrabutylammonium salt of 2-nitroimidazole (0.481 g, 1.36 mmol) was used; the reaction was started at −50 °C; and the reaction mixture was allowed to warm to RT in 18 h. The crude product (*α*/*β* = 2/1, by ^1^H NMR) was flash chromatographed (hexanes/EtOAc = 2/1, α-**19**: *R*_*f*_ = 0.25; *β*-**19**: *R*_*f*_ = 0.21) to yield 0.536 g mixture (81%, *α*/*β* = 2/1) of α- and *β*-**19**. The anomers were separated by flash chromatography (CH_2_Cl_2_/EtOAc = 20/1; *α*: *R*_*f*_ = 0.42; *β*: *R*_*f*_ = 0.36) using a long column to yield homogenous anomers and mixture of anomers.

*α*-**19**: Oil, which decomposed at room temperature within a few days, but it was more stable at 4 °C. When *α*-**19** was crystallized from C_2_H_4_Cl_2_/*i*-Pr_2_O by slowly cooling from RT to −18 °C, a white powder was obtained, which contained after drying at 0.5 mbar/RT for 10 h 0.05 mol% of *i*-Pr_2_O; m.p.: 63–65 °C (powder became glassy); this powder was ideal for storage at 4 °C and handling. [*α*]_D_^20^ = −9.78 g cm^2^ (*c* = 1.15, acetone); ^1^H NMR (400.13 MHz, CDCl_3_): *δ* = 7.85–7.80 (m, 2H, H^ar^), 7.66–7.62 (m, 2H, H^ar^), 7.57–7.52 (m, 1H, H^ar^), 7.41–7.33 (m, 4H, H^ar^), 7.32 (d, *J* = 1.0 Hz, 1H, H^im^), 7.13 (d, *J* = 1.0 Hz, 1H, H^im^), 6.62 (d, *J* = 6.6 Hz, 1H, 1′-H), 5.43 (d, *J* = 6.6, 0.7 Hz, 1H, 3′-H), 4.74 (td, *J* = 3.0, 1.0 Hz, 1H, 4′-H), 4.37 (AB part of ABX system, *J*_AB_ = 11.4 Hz, *J*_AX_ = *J*_BX_ = 3.0 Hz, 2H, 5′-H), 3.05 (td, *J* = 15.5, 6.6 Hz, 1H, 2′-H), 2.48 (d, *J* = 15.5 Hz, 1H, 2′-H), 2.45 (s, 3H, CH_3_) ppm; ^13^C NMR (100.61 MHz, CDCl_3_): *δ* = 165.7 (CO), 145.6 (Cq^tos^), 143.7 (Cq^im^), 133.9 (HC), 132.43 (Cq), 130.1 (2 HC^tos^), 129.5 (2 HC^ar^), 128.6 (2 HC^ar^), 128.4 (Cq^ar^), 128.2 (HC^im^), 127.9 (2 HC^ar^), 122.2 (HC^im^), 91.4 (C-1′), 86.1 (C-4′), 74.6 (C-3′), 69.0 (C-5′), 41.1 (C-2′), 21.7 (CH_3_^tos^) ppm; and IR (ATR, NMR sample): $\bar{\nu }$ = 2971, 1709, 1535, 1476, 1355, 1270, 1240, 1175, 1092, 1075 cm^−1^.

*β*-**19**: [*α*]_D_^20^ = −16.89 g cm^2^ (*c* = 1.06, acetone); m.p.: 90 °C (decomp., CH_2_ClCH_2_Cl/*i*-Pr_2_O, solution not heated above 50 °C); ^1^H NMR (400.13 MHz, CDCl_3_): *δ* = 8.03–7.96 (m, 2H, H^ar^), 7.82–7.75 (m, 2H, H^ar^), 7.63–7.57 (m, 2H, H^ar^), 7.60 (d, *J* = 1.0 Hz, 1H, H^im^), 7.50–7.43 (m, 2H, H^ar^), 7.38–7.32 (m, 2H, H^ar^), 7.17 (d, *J* = 1.0 Hz, 1H, H^im^), 6.78 (dd, *J* = 7.6, 5.6 Hz, 1H, 1′-H), 5.42 (td, *J* = 6.6, 2.3 Hz, 1H, 3′-H), 4.48–4.37 (m, 3H, 5′-H and 4′-H), 2.99 (ddd, *J* = 14.3, 5.6, 2.3 Hz, 1H, 2′-H), 2.43 (s, 3H, CH_3_), 2.41 (ddd, *J* = 14.3, 7.6. 6.6 Hz, 1H, 2′-H) ppm; ^13^C NMR (100.61 MHz, CDCl_3_): *δ* = 165.9 (CO), 145.7 (C_q_^tos^), 144.0 (Cq^im^), 133.9 (HC^Ph^), 132.2 (Cq^tos^), 130.2 (2C, HC^tos^), 129.7 (2C, HC^tos^), 128.96 (HC^im^), 128.7 (Cq^Ph^), 128.6 (2C^Ph^), 127.9 (2C^Ph^), 121.8 (C^im^), 88.8 (C-1′), 83.2 (C-4′), 74.4 (C-3′), 68.5 (C-5′), 40.1 (C-2′), 21.7 (CH_3_^tos^) ppm; and IR (ATR, NMR sample): $\bar{\nu }$ = 1713, 1544, 1352, 1279, 1174, 1096 cm^−1^.

### *Preparation of 3*-*O*-*benzoyl*-*2*-*deoxy*-*5*-*O*-*tosyl*-*D*-*ribofuranosyl chlorides and their conversion to α*- *and β*-*19 by general procedure C*

A mixture of 0.495 g methyl glycosides *α*- and *β*-**18** (1.22 mmol) was transformed via glycosyl chlorides (general procedure A) into nucleosides *α*- and *β*-**19** by general procedure C. The crude product (*α*/*β* = 1/5, by ^1^H NMR) was flash chromatographed (hexanes/EtOAc = 2/1) using a long column to yield 0.066 g nucleoside *α*-**19** (14%) and 0.327 g *β*-**19** (69%).
